# Brain Barrier Aging is Associated with Changes in Brain Plasticity and Decline in Processing Speed: A Proteomic Study of Human Cerebrospinal Fluid

**DOI:** 10.1002/alz.087127

**Published:** 2025-01-03

**Authors:** Fanny Elahi

**Affiliations:** ^1^ Departments of Neurology and Neuroscience, Icahn School of Medicine at Mount Sinai, New York, NY USA; ^2^ James J. Peters Department of Veterans Affairs Medical Center, Bronx, NY USA

## Abstract

**Background:**

Protective brain barriers, such as blood‐brain barrier, become dysfunctional with age. The BBB is a dynamic and selective barrier, gating the passage of molecules and cells to and from the brain. The function of this barrier is critical for the maintenance of brain homeostasis. BBB dysfunction has been reported in aging and disease states, with downstream consequences on long‐range neuro‐glial networks subserving emotions, cognition, movement, and sensations. The molecular mediators of the effect of BBB leakage on neuro‐glial networks is not well understood.

**Methods:**

Using large‐scale proteomics and targeted ELISA assays to interrogate over 7000 proteins in cerebrospinal fluid (CSF) of highly phenotyped aging older adults, we interrogated signatures of BBB dysfunction in aging and AD. Our analytical workflow consisted of principal component analysis, linear modeling, and ontology analyses.

**Results:**

We identified distinct CSF‐proteomic signatures for leaky BBB and demonstrate associations with markers of synaptic dysfunction and slowed processing speed. We found several associations between BBB leakage and upregulation in pathways classically thought to be neurodevelopmental including Ephrin and neuropilin signaling. We validated our findings in an independent Alzheimer’s disease dataset.

**Conclusion:**

This study presents a multivariate approach for investigating large‐scale omics data and provides insights into the potential mechanisms for vascular cognitive aging, with a focus on aging of the brain’s barriers.

